# Mitochondrial stress: a key role of neuroinflammation in stroke

**DOI:** 10.1186/s12974-024-03033-7

**Published:** 2024-02-06

**Authors:** Ling Gao, Li Peng, Jian Wang, John H. Zhang, Ying Xia

**Affiliations:** 1 Department of Neurosurgery, Xiangya School of Medicine, Affiliated Haikou Hospital, Central South University, Haikou, 570208 China; 2 Department of Physiology and Pharmacology, School of Medicine, Loma Linda University, Loma Linda, CA 92354 USA; 3 Department of Neurosurgery and Anesthesiology, Loma Linda University Medical Center, Loma Linda, CA 92354 USA; 4 Department of Ophthalmology, Xiangya School of Medicine, Affiliated Haikou Hospital, Central South University, Haikou, 570208 China

**Keywords:** Mitochondrial stress, Mitophagy, Neuroinflammation, Stroke

## Abstract

Stroke is a clinical syndrome characterized by an acute, focal neurological deficit, primarily caused by the occlusion or rupture of cerebral blood vessels. In stroke, neuroinflammation emerges as a pivotal event contributing to neuronal cell death. The occurrence and progression of neuroinflammation entail intricate processes, prominently featuring mitochondrial dysfunction and adaptive responses. Mitochondria, a double membrane-bound organelle are recognized as the “energy workshop” of the body. Brain is particularly vulnerable to mitochondrial disturbances due to its high energy demands from mitochondria-related energy production. The interplay between mitochondria and neuroinflammation plays a significant role in the pathogenesis of stroke. The biological and pathological consequences resulting from mitochondrial stress have substantial implications for cerebral function. Mitochondrial stress serves as an adaptive mechanism aimed at mitigating the stress induced by the import of misfolded proteins, which occurs in response to stroke. This adaptive response involves a reduction in misfolded protein accumulation and overall protein synthesis. The influence of mitochondrial stress on the pathological state of stroke is underscored by its capacity to interact with neuroinflammation. The impact of mitochondrial stress on neuroinflammation varies according to its severity. Moderate mitochondrial stress can bolster cellular adaptive defenses, enabling cells to better withstand detrimental stressors. In contrast, sustained and excessive mitochondrial stress detrimentally affects cellular and tissue integrity. The relationship between neuroinflammation and mitochondrial stress depends on the degree of mitochondrial stress present. Understanding its role in stroke pathogenesis is instrumental in excavating the novel treatment of stroke. This review aims to provide the evaluation of the cross-talk between mitochondrial stress and neuroinflammation within the context of stroke. We aim to reveal how mitochondrial stress affects neuroinflammation environment in stroke.

## Introduction

Stroke is a clinical syndrome characterized by an acute and focal neurological deficit, caused by the occlusion or rupture of cerebral vessels [1]. Stroke is classified into hemorrhagic stroke caused by blood vessel rupture, and ischemic stroke resulted from blood clot-induced obstruction of cerebral blood flow. Further categorizations within hemorrhagic stroke include intracerebral hemorrhage (ICH) and subarachnoid hemorrhage (SAH) [1, 2]. Notably, over 60% of all incident strokes is ischemia stroke, which constitutes the largest population of stroke [3]. It is noteworthy that stroke ranks as the second leading cause of death in the world. According to a report in 2019 by World Stroke Organization, stroke claimed the lives of over 6.50 million annually, and accounted for at least 1.43 million disability-adjusted life lost [4, 5]. Unfortunately, even a few effective medical or surgical therapies have been demonstrated to enhance the prognosis of stroke patients, survivors of stroke commonly contend with sequelae of neurological impairments and psychiatric disorders, exerting a profound impact on their daily functionality and occupational capacity. Therefore, dedicated research efforts into the pathophysiological mechanisms of stroke are essential for developing new treatments and ultimately improving outcomes for these patients.

Neuroinflammation is the remarked event of stroke and also contributes to the neural cell death [5–7]. In stroke, the pathological condition increases the permeability of blood–brain barrier (BBB) [8], facilitating the infiltration of deleterious substances into neuronal microenvironment. Subsequently, microglia, a specific type of brain resident macrophage, are activated by the damage-associated molecular patterns (DAMPs) released from impaired neurons [9]. And these cells transformed to into the classical M1 phenotype and produce lots of inflammation-related cytokines to recruit other immune cells [10, 11], thereby fostering a neuroinflammation microenvironment in the brain. Similarly, the activation of other glia, such as astrocytes and oligodendrocytes, modulates the production and release of inflammatory cytokines in stroke. Indeed, neuroinflammation plays a pivotal role in causing brain tissue damage after both ischemic and hemorrhagic strokes. Understanding the mechanisms of neuroinflammation is essential for developing effective therapies to improve stroke outcomes.

As cellular powerhouses, mitochondria have garnered significant attention and reevaluation. Recent research has unveiled a profound correlation between mitochondrial dynamics and the pathophysiology of stroke [12, 13]. Mitochondrial dysfunction and adaptive alteration can affect the occurrence and development of neuroinflammation [14]. Dysfunctional mitochondrial dynamics inciting innate immune responses in both resident and infiltrating cells, such as microglia, astrocytes and oligodendrocytes, encompassing calcium-dependent immune activation, phosphorylation of transcription factors, cytokine secretion, organelle translocation, and the release of mitochondrial damage-associated molecular patterns (mDAMPs), even cell death [14, 15], impacting the outcome of stroke. Meanwhile, under neuroinflammation situation, the activation of microglia/astrocytes release various pro-inflammatory factors like IL-6, IL-1β, TNF-α and chemokine [16–18], promote the secretion of inflammasome [19]. Inflammasome and pro-inflammation factors associate with mitochondrial dysfunction lead to release reactive oxygen species (ROS) and mDAMPS which, in turn, aggravate inflammation consequently [20]. Eventually, the synergistic effect of neuroinflammation and mitochondrial stress cause brain edema, brain–blood barrier disruption and cell death [21, 22]. Therefore, understanding the correlation between mitochondrial and neuroinflammation in stroke pathogenesis is essential in excavating the novel treatment of stroke. In this review, we provide the evaluation of the cross-talk between mitochondrial stress and neuroinflammation, focusing on stroke. We aim to reveal how mitochondrial stress affects neuroinflammation environment in stroke.

## Mitochondria and mitochondrial stress

Mitochondrion, a double membranes-bound organelle, is divided into four suborganellar compartments: outer mitochondrial membrane (OMM), inner mitochondrial membrane (IMM), intermembrane space between OMM and IMM (ISM) and mitochondrial matrix surrounded by IMM [23]. The matrix of human mitochondria contains mitochondrial circular DNA encoding 2 ribosomal RNAs (rRNA) including 12S and 16S rRNA, 13 proteins and 22 transfer RNAs (tRNA) [24]. Although mitochondrial genome is capable of encoding several factors required for the organelle activity, mitochondria still depend on nuclear genome. Most of mitochondrial proteins are encoded by nuclear genome. For example, only 11 proteins of proteins of oxidative phosphorylation (OXPHOS) are encoded by mitochondrial genome whereas 1300 OXPHOS-related proteins are nuclear gene products [25].

These nuclear genome-encoded proteins are transferred to mitochondria based on mitochondrial protein import pathways. Mitochondrial protein import is a conserved cellular process. In this process, most of mitochondrial proteins, which bind to some chaperones such as heat shock protein (HSP) 70 and 90, can be recognized by the translocase of the outer membrane (TOM) complex via their mitochondrial targeting pre-sequences (MTS). TOM complex is a symmetrical dimer that consists of 10 membrane protein subunits including TOM5, TOM6, TOM7, TOM22 and TOM40, which forming a shallow funnel on OMM [26–30]. After imported translocated across OMM, mitochondrial proteins are directed to the presequence translocase of the inner membrane complex (TIM23 complex), followed by the insert into IMM through the translocase of IMM (TIM22) complex, or the import to mitochondrial matrix by the mitochondrial import complex (MIM complex) [31].

**Fig. 1:**
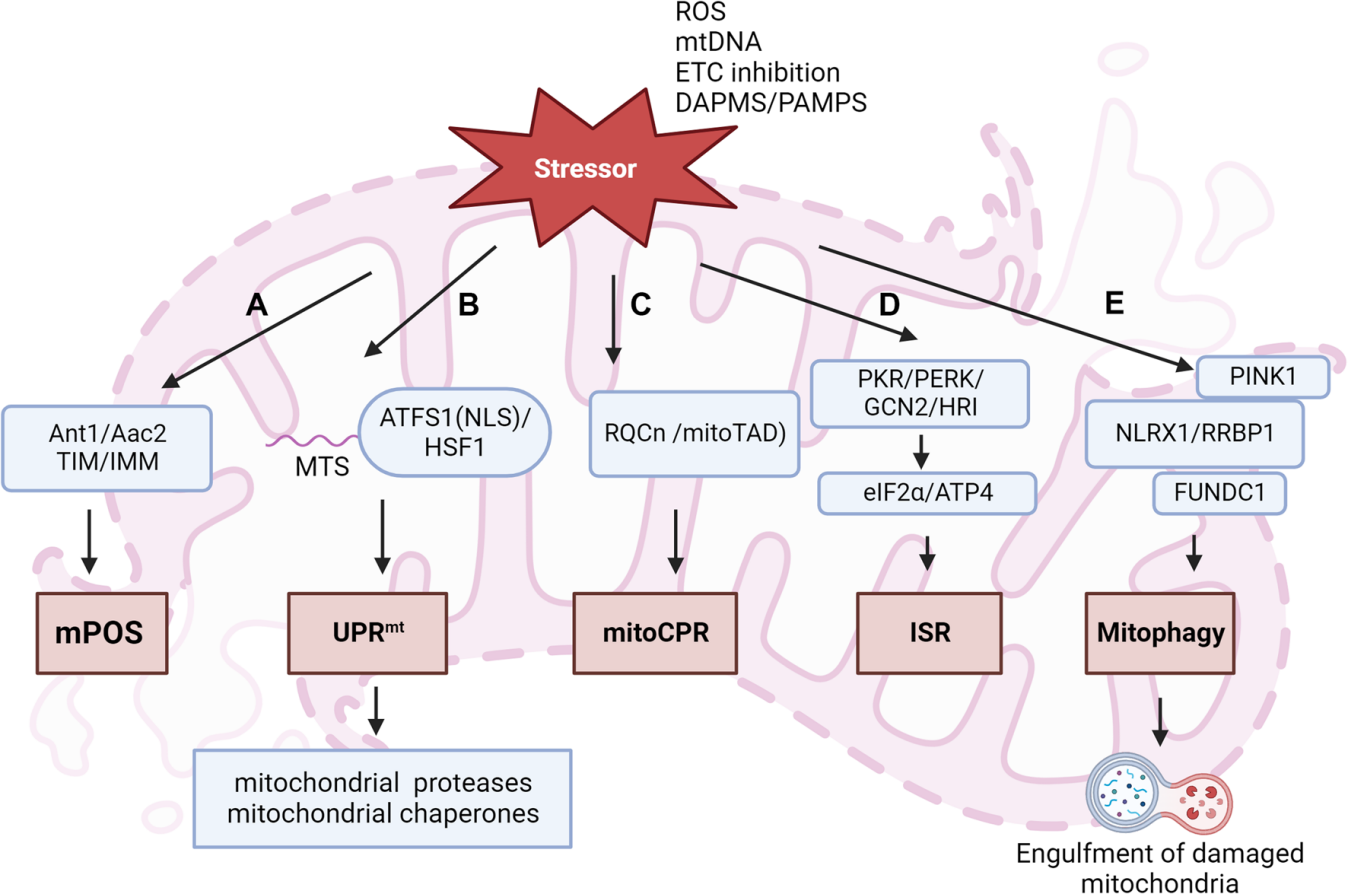
Mitochondrial stress response pathways. Environmental disturbance or impairment result in a decrease in mitochondrial stressor (ROS, mtDNA, ETC inhibition and PAMPs/DAPMs). Mitochondrial stress is activated to respond those stressors. **A** The mutant form of Ant1/Aac2 impairs the structural stability of TIM22, which in turn inhibits the biogenesis of TIM23 and induces instability in the IMM. This disruption results in proteostasis stress and triggers mitochondrial precursor overaccumulation stress (mPOS). **B** The unfolded protein response in mitochondria (UPRmt) is regulated by competing organelle targeting sequences within the transcription factor ATFS-1. When ATFS-1 is imported into the mitochondrial matrix via the MTS, it is subjected to degradation. However, in cases of mitochondrial dysfunction where ATFS-1 cannot be imported, it is redirected to the nucleus through the nuclear localization sequence (NLS). There, it activates the transcription of mitochondrial chaperones and proteases. **C** Under normal conditions, mitochondrial protein import undergoes rigorous monitoring by various quality control mechanisms, including ribosomal quality control (RQC) and mitochondrial translocation-associated degradation (mitoTAD). However, if RQC and mitoTAD are impaired due to mitochondrial dysfunction, a response known as mitochondrial compromised protein import response (mitoCPR) occurs within the cells. **D** Mitochondria transmit stress signals, activating the integrated stress response (ISR). This classic pathway involves four stress-activated kinases: PKR, PERK, GCN2, and HRI. They phosphorylate eIF2α during cellular stress, inhibiting protein biosynthesis and activating ATP4 transcription, thus promoting adaptive and apoptotic gene expression in response to stress. **E** Recognition and selective degradation of damaged mitochondria is mediated by mitophagy. The kinase PINK1 is stabilized specifically on damaged mitochondria where it recruits the ubiquitin ligase Parkin, which ubiquitinates multiple mitochondrial outer membrane proteins. Ubiquitinated mitochondria are then engulfed by autophagosomes and trafficked to lysosomes where they are degraded

Mitochondrial protein import involves the communication to multiple signal pathways in the cytoplasm and organelles [32]. Meanwhile, mitochondria provide the large amounts of energy for organelle activity in cellular processes [32]. Thus, the cross-talks of mitochondria and other organelle cause itself easy to respond to cellular stress. A series of mitochondrial response to stressors is called ‘mitochondrial stress’ (Fig. 1). The moderate mitochondrial stress promotes the cellular adaptive protection to resist harmful stressors. While the continuous mitochondrial stress impairs cells and tissues. Numerous constituents within mitochondria, along with metabolic byproducts, can function as mDAMPs. When released into the cytosol or extracellular environment after mitochondria injured, these mDAMPs trigger pro-inflammatory cytokine production and the recruitment of immune cells [15], which subsequently contributes to oxidative stress, inflammation, energetic impairment cell death and tissue injury [33, 34].

### Mitochondrial stress: stressor, stress response and communication mode

#### Stressors

Environmental disturbance or impairment result in a decrease in mitochondrial membrane protein (MMP) and misfolded protein accumulation in mitochondria. Mitochondrial stress is activated to respond these alterations. We introduce some of common stressors of mitochondrial stress.

ROS is the biomarker of oxidative stress, which can be produced in various location within the cell from plasma membrane to nucleus [35]. Mitochondrial ROS (mtROS) play a major role in the physiological or pathological condition, although ROS can be generated in many organelles [36]. The results of mtROS accumulation in mitochondria relate to the decrease of MMP, release of cytochrome C and leak of mitochondrial DNA (mtDNA) [37]. The ROS-induced release of cytochrome C and mnntDNA may involve gasdermin D (GSDMD), a pore-forming protein. A current report by Weindel et al. concluded mitochondrial ROS caused GSDMD to direct to mitochondrial membranes. which promoted mitochondrial content outflow to the cytoplasm [38]. Moreover, mitochondrial ROS is involved in the expression of nuclear genes. Peroxisome proliferator-activated receptor γ coactivator 1α (PGC-1α) is associated with the role of ROS in nuclear genome. PGC-1α is a transcript coactivator expressed in the tissues with high energetic demands [39]. PGC-1α is the core factor for oxidative stress and inflammation. On the one hand, PGC-1α interacts nuclear factor kappa B (NF-κB) p65 subunit to block its transcriptional activity on pro-inflammation genes, thereby playing the anti-inflammatory role [40]. As the negative feedback, NF-κB p65 directly inhibits PGC-1α via the binding between them [41]. On other hands, PGC-1α inhibits ROS accumulation in mitochondria via triggering the expression of ROS detoxification proteins such as superoxide dismutase 2 (SOD2) and glutathione peroxidase 1 (GPX1) [42]. The increased ROS activates NF-κB p65 to induce PGC-1α dysfunction, thereby causing the decreased expression of SOD2 and GPX1.

mtDNA is easily affected by environmental disturbance or impairment. mtDNA mutation contributes to mitochondrial stress. Zhang et al. showed mtDNA increase was associated with UPR^mt^ in the *C. elegans* model [43]. The intriguing finding indicate mtDNA mutation has the potential of inducing stress signals via UPR^mt^. mtDNA mutation blocks the mitochondria–nuclear communication. Recently, a mouse model with mtDNA mutation develops the specific profile that mtDNA causes the depletion of total cellular nucleotides, suggesting the role of mtDNA mutation in nuclear genome stability [44].

ROS is the common byproduct of OXPHOS. There is a mature regulator mechanism to prevent ROS accumulation based on ROS detoxification proteins. For example, cells performing OXPHOS form the phenotype with the upregulated ERK5 that binds to the promoter of NRF2 as a transcript factor [45]. However, uncoupling of OXPHOS impairs the adjusting in cells and affects mitochondrial homeostasis. In response to the impairment of OXPHOS uncoupling, cells show the adaptive phenotype with the compensatory elevation in PGC-1α, COXIV and ANT3 and the rise in glycolytic metabolism but the reduction in cellular growth [46, 47]. OXPHOS uncoupling inhibits the translocation of mitochondrial protein from nuclear to mitochondria, which further induces mitochondrial unfolded protein response (UPR^mt^). Thus, OHPHOS uncoupling can cause UPR^mt^ activation.

Electron transport chain (ETC) inhibition impairs OXPHOS and then triggers UPR^mt^. Mitochondria with ETC inhibition can develop the increased mitochondrial calpain 1 that has the capability of cleaving apoptosis inducing factor, a dual-function protein that can remove ROS in the physiological condition or translocate to nuclear to mediated caspase-dependent and caspase-independent apoptosis when sensing apoptosis stimuli [48]. A dominant mutation occurring in IMS protein is a cause of ETC-related mitochondrial stress. Mitochondrial coiled-coil-helix-coiled-coil-helix domain-containing 10 (CHCHD10) plays the core role of ETC integrity and mitochondrial structure via binding to mitochondrial nuclear retrograde regulator 1 that interacts with complex IV, the last electron receptor of ETC [49]. CHCHD10 mutation contributes to mitochondrial dysfunction with ETC inhibition [50]. Particularly, OMA1 is activated to induce mitochondrial fission, UPR^mt^ and integrated stress response (ISR) when the dominant p.G58R mutation in CHCHD10 occurs [50].

#### Stress response

##### Mitochondrial precursor overaccumulation stress (mPOS)

Mitochondrial protein import is exquisitely modulated at multiple aspects, which causes mitochondria easier to develop the complex and multifaceted physiological consequences when exposed to stress. Some of pathogenic stressors that indirectly affect the core protein import inhibit cell adaption to stress through interfering the proteostasis network in the cytoplasm [51], which results in a decrease in protein import that further cause mitochondrial precursor overaccumulation in the cytoplasm. Cells adjust itself survival and death in response to mitochondrial precursor overaccumulation due to severe proteostasis stress, which is called ‘mPOS’ [52]. Ant1, an IMM protein related to ATP/ADP exchange, plays the key role in mPOS. The mutant of Ant1 is capable of inducing mPOS via disturbing the assembly and stability of mitochondrial membranes. The misfolded variants of Aac2, the homologue of Ant1 in yeast, can form aggregates to cause proteostasis stress [53]. Also, it impairs the structural stability of TIM22, which subsequently leads to the defect of TIM23 preprotein translocase [53]. The Aac2-induced instability of IMM and proteostasis stress triggers mPOS. Interestingly, human Ant1 activates a defense mechanism against mPOS in addition to the role in the induction of misfolded protein overaccumulation [54]. The overexpression of mutant Ant1 results in aggregate formation to activate protein degradation, autophagy and cell death, which is assumed as the anti-mPOS mechanism [54].

##### Mitochondrial unfolded protein response (UPR^mt^)

Mitochondrial unfold protein response (UPR^mt^) is a stress response that protects mitochondrial function by stop protein translation and degrade unfolded proteins when mitochondria is impaired by unfolded proteins [55]. UPR^mt^ characterized by the activation of molecular chaperones and proteases is activated to decrease protein toxicity of mitochondrial stress. In UPR^mt^, activated chaperones are used for protein folding and proteases are for protein degradation [56]. The inefficient import of activating transcription factor associated with stress 1 (ATFS1) is the key event of UPR^mt^. It has been determined to co-ordinate the protective transcription response in *yeast*, *C. elegans*, *and* mammal during UPR^mt^. ATFS1 structure contains MTS mediating protein import in the normal condition and nuclear localization signal (NLS) promoting translocation to nuclear under stress [57]. When stressed, ATFS1 import is inhibited and then it, because of NLS, transfers to the nuclear for the transcription activation of targets, including Dnj10, HSP60, skn1, gpd2, TIM23 and TIM17 [57]. UPR^mt^ has the ability to upregulate the efficiency of protein import to prolong mitochondrial lifespan. Intriguingly, ATFS1-dependent activation of UPR^mt^ can promote mitochondrial import, despite its membrane potential is reduced [58], suggesting a positive feedback between UPR^mt^ and relatively healthy mitochondria that these mitochondria transport signals to activate UPR^mt^ which enhance the import of reversive protein for the assembly and biogenesis of mitochondria. In human, UPR^mt^ is regulated by the homologue of ATFS-1 including activating transcription factor 4 (ATF4) and ATF5.

In addition to ATFS1, heat shock transcription factor 1 (HSF1) is also required for UPR^mt^. In UPR^mt^, ATFS1 is used to activate the transcription of nuclear genes that contribute to cell fitness involved in metabolism and proteases, while HSF1 plays the role of inducing mitochondrial chaperones. In response to mitochondrial stress, HSF1 is upregulated to bind constitutively to the promoters of genes encoding mitochondrial chaperones, including HSP60, HSP10 and mitochondrial HSP70 [59]. How does mitochondria transmit the stress signal to HSF1 during UPR^mt^? A recent study showed the potential mechanism involving mtROS and mitochondrial precursor overaccumulation (mtPO). During mitochondrial stress, mtROS released to the cytoplasm oxidates cytosolic HSP40, a co-chaperon of HSP70, which leads to HSP70 recruitment in mtPO that promotes the release of HSF1 to translocate in the nuclear [60]. HSF1-mediated transcription activation appears to stop at the end of the acute stress response, and this protein activated by acute stress cannot induce the transcription of its targets under chronic stress, which suggests HSF1 is required for acute stress rather than chronic or continuous stress [61].

##### Mitochondrial compromised protein import response (mitoCPR)

In the normal condition, mitochondrial protein import is under rigorous monitor by various mechanism of quality of control, such as ribosomal quality control [62] and mitochondrial translocation-associated degradation [63]. However, the common maintaining mechanisms are not enough to meet the overaccumulation of mitochondrial protein during stress. The *Yeast* develops compensatory and acute mechanism to mitigate protein import stagnation in the pathological condition, which may be not conversed in higher eukaryotes. In *yeast*, Pdr3, a transcription factor, induces Cis1 expression that is capable of recruiting Msp1 translocation to TOM complex, which promote the stalled import protein to be degraded in the cytoplasm [64]. The acute mechanism responding to mitochondrial stress is called ‘mitoCPR’. It remains unclear whether mitoCPR occurs in human since there is no obvious ortholog of Pdr3. Moreover, it needs to be uncovered whether mitoCPR function as a pre-order mechanism to respond to the inefficient protein import before mPOS and UPR^mt^ [65].

##### Integrated stress response (ISR)

Mitochondrial stress is not a single mechanism. It demands the co-ordination of multiple cellular response and signaling pathways. Mitochondria can transmit stress signals to trigger in ISR. The classical ISR pathway consists of four stress-activated kinases, including Protein kinase R, Protein kinase R-like endoplasmic reticulum kinase, general control nonderepressible 2 and heme-regulated inhibitor (HRI) that have the ability to phosphorylate eukaryotic initiation factor 2α (eIF2α) when stressed [66]. Phosphorylated eIF2α inhibits the whole biosynthesis of protein via its activity in transcription inhibition. Meanwhile, it also activates ATP4 transcription to promote the expression of adaptive and apoptotic genes in response to stress [67]. Recent insight into integrated stress response concludes stressed mitochondria leads to the PHB2-STOML2 complex disintegration, thereby activating OMA1 to cleave DELE1 that binds to HRI after relayed to the cytoplasm and induce HRI-mediated eIF2α phosphorylation [67, 68].

##### Mitophagy

Mitophagy is a cellular process wherein mitochondrial-derived vesicles selectively engulf specific mitochondrial components and transport them to lysosomes or peroxisomes for degradation [69]. This quality control mechanism is pivotal for maintaining mitochondrial content and metabolic homeostasis [69, 70]. In cells, mitochondrial stress and mitophagy are two axes to resist the pathological condition. The former is used to improve the inefficient protein import to mitochondria, and the latter contributes to removing impaired mitochondria [70]. An imbalance between these processes can result in the accumulation of dysfunctional mitochondria, heightened oxygen consumption, and excessive production of ROS [70]. Ultimately, this imbalance can lead to cellular degeneration and activation of cell death pathways [70]. The co-ordination of mitochondrial stress and mitophagy is observed in recent studies. For example, mitophagy can promote global inhibition to protein biogenesis during mPOS via removing the impair mitochondria. In response to mPOS, cells trigger LC3 lipidation to activate mitophagy via NLRX1/RRBP1 complex [71]. There is the co-ordination between mitophagy and UPR^mt^. Myocardial mitochondria show the co-ordination between mitophagy and UPR^mt^ to protect mitochondria from stress [72]. In *C. elegans,* FNDC1, an ortholog of FUN14 domain containing 1 (FUNDC1), and ATFS1 [73] mediate the cross-talk between mitophagy and UPR^mt^. Dysfunction of FNDC1-mediated mitophagy contributed to impaired mitochondria accumulation that cause UPR^mt^ activation [73]. In turn, mitochondrial stress is also able to induce FUNDC1-mediated mitophagy [74].

#### Communication mode

Nuclear genome encodes most of mitochondrial protein. The forward signaling from nuclear to mitochondria is required for the homeostasis and integrity of mitochondria. Mitochondria also are able to influence the expression of nuclear genes via various communication mediators, which is named ‘mitochondrial retrograde signaling’. Mitochondrial retrograde signaling is responsible for transmitting mitochondrial stress to cells. We conclude common communication molecules in mitochondrial retrograde signaling.

ROS is not just a stressor, but also serves as a communication molecule of mitochondrial stress. ETC inhibition due to OXPHOS uncoupling-related proton leak to promote one electron reduction of oxygen to superoxide, which contributes to the production and release of mitochondrial ROS [75]. Then, ROS, as the signal, activates NRF2-related UCP1 expression (a protein for mitochondrial uncoupling) [76–78] and ATF family-mediated UPR^mt^ [79, 80], and recruits Parkin to mitochondria for mitophagy [81, 82].

In multicellular organisms, local stress can be transmitted to the distant cell/tissue to induce systemic regulation for the whole metabolism. The non-autonomous response is mediated by a series of factors named ‘mitokine’. Fibroblast growth factor 21 (FGF21) and growth differentiation factor 15 (GDF15) are two mitokines to be most discussed. FGF21 and GDF15 can be used for the biomarkers in the first-line diagnosis of mitochondrial disease [83, 84]. FGF21 and GDF15 show the different roles in metabolic adaption to mitochondrial stress although both of them are capable of transmitting stress signals.

FGF21 is upregulated in mitochondrial stress. Stressors such as OXPHOS uncoupling increase FGF21 expression to transmit ISR [85]. Mitochondrial dysfunction caused the upregulation of *Fgf21* mRNA expression in nucleus [86], upregulation of FGF21 is instrumental in cardio-protection and mitochondrial quantity control. FGF21 is able to activate AMPK/FOXO3/SIRT3 pathway to inhibit the mitochondrial dysfunction in cardiomyocytes [87]. Li et al. showed depletion of FGF21 contributes to the production of mitochondrial ROS [88]. They further determined mitochondrial fusion was activated by FGF21 based on AMPK pathway. FGF21 plays the role in systemic metabolism and mitochondrial stress via interacting with β-klotho, an obligatory co-receptor for FGF21. In cells, the C terminal of FGF21 binds to β-klotho to activate fibroblast growth factor receptor, which promotes cell signaling transduction [89]. Notably, FGF21 shows a characteristic with diurnal oscillations. Circulating FGF21 reaches the peak at the early morning, and then decrease to the minimum at the afternoon [90], suggesting the dynamic role of FGF21 in metabolism regulation.

Similar to FGF21, GDF15 is increased by mitochondrial stressors. In nucleus *Crif-1* increase *Gdf15 RNA* expression via UPR^mt^/ATF4/CHOP pathway [91]. GDF15 can be induced by ETC inhibition to mediate systemic metabolic flexibility related to glycolipid metabolism [92]. Also, exercise leads to the dramatic upregulation of plasma GDF15 [93]. GDF15 is expressed in multiple tissues, such as lung, heart, brain, kidney, liver, adipose, skeletal muscle and gastrointestinal system. There is a specific receptor of GDF15 expressed, called as ‘GDNF-family receptor α-like (GFRAL)’ in the hindbrain. GDF15 is capable of interacting with GFRAL to orchestrate its role in lipid metabolism. Interestingly, GDF15 also shows the diurnal rhythm in the body. Circulating GDF15 in the day is higher than that in the night in a UCP1-tg mice developing mitochondrial uncoupling [92].

## Cross-talk between mitochondrial stress and neuroinflammation in stroke

**Fig. 2:**
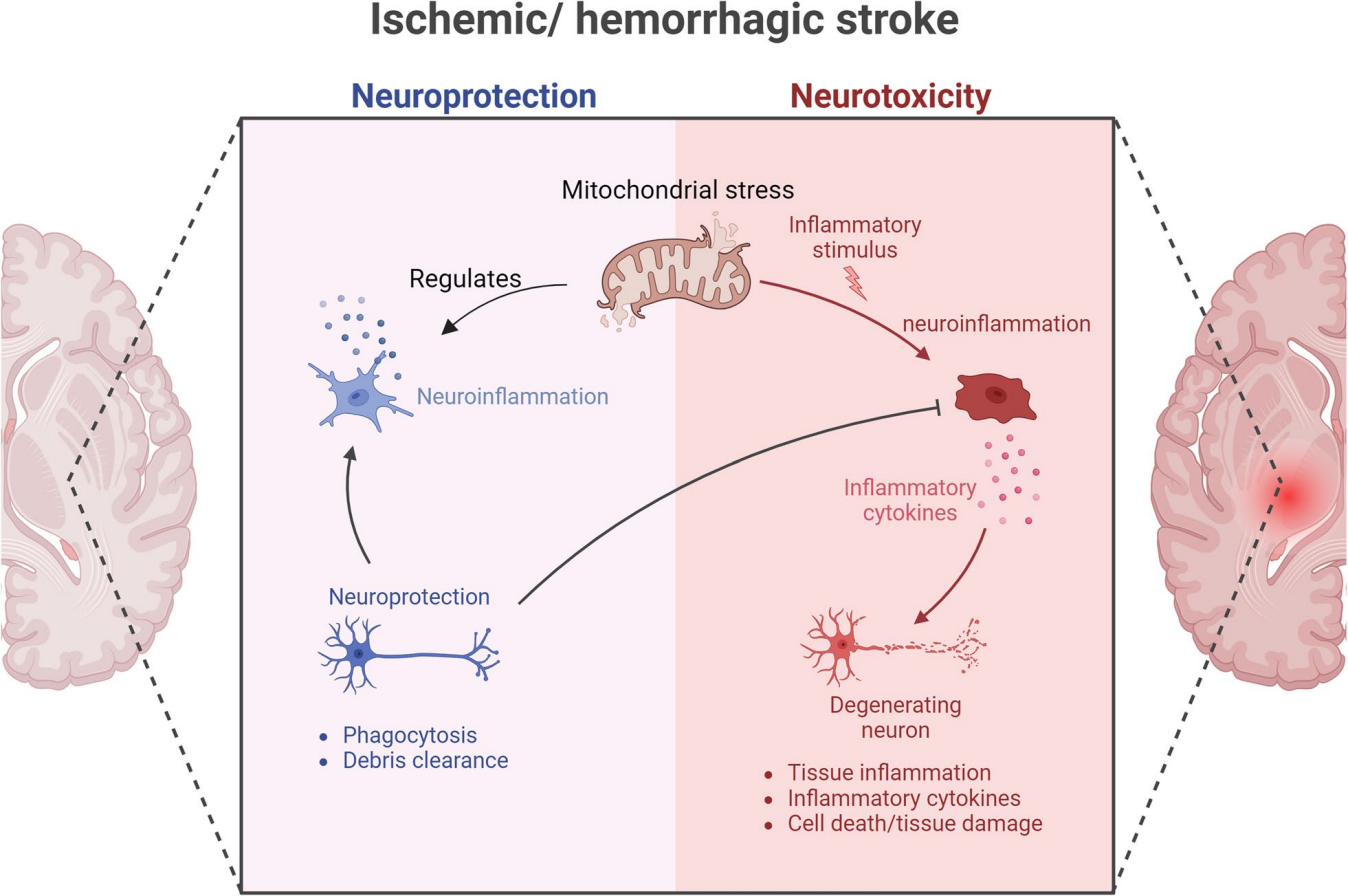
Cross-talk between mitochondrial stress and neuroinflammation in stroke. Mitochondrial stress serves as an adaptive mechanism aimed at mitigating the stress induced by the import of misfolded proteins, which occurs in response to a stroke. The significance of mitochondrial stress in the pathogenesis of stroke is highlighted by its ability to interact with neuroinflammation. The impact of mitochondrial stress on neuroinflammation varies depending on its severity. Moderate mitochondrial stress can modulate neuroinflammation, enabling cells to better withstand harmful stressors and protect neurons. In contrast, sustained and excessive mitochondrial stress promotes neuroinflammation, leading to the release of pro-inflammatory cytokines, which negatively impacts cellular and tissue integrity

Neuroinflammation, the inflammatory response following neuronal injury, plays a pivotal role in stroke pathogenesis [9, 94]. Under mitochondrial stress, diverse cell types, such as astrocytes, microglia, endothelial cells, and leukocytes, release pro-inflammatory agents, including chemokines, cytokines, and enzymes. These actions collectively contribute to damage in the cerebral parenchyma [6]. Notably, accumulating evidence highlights the dual nature of these cells in inflammation, with their effects being either advantageous or harmful, depending on the timing of pathway activation or inhibition [95]. Mitochondrial stress after stroke is capable of interacting with neuroinflammation lead to neurovascular unit (NVU) dysfunctions, including neuron death, BBB disruption, and neuroinflammation, and affect the outcome of stroke [13]. The turbulences of mitochondrial function include increased fission, deficient fusion, and impaired or excessive mitophagy [12]. However, the role of mitochondrial stress in neuroinflammation and stroke is still under debate and we will discuss whether mitochondrial stress promotes or inhibits neuroinflammation in stroke in this section (Fig. 2).

### Mitochondrial stress modulates neuroinflammation to resist stroke

Although much of the research described previously has characterized mitochondrial stress as a deleterious process in stroke-induced brain injury, there is also evidence pointing to its neuroprotective effects.

Mitochondrial stress has been employed as a strategy to mitigate excessive damage resulting from neuroinflammation. A study by Zhu et al. demonstrated the activation of microglia and the UPR^mt^ (reflecting mitochondrial stress) are involved in the pathogenic mechanism underlying hydrocephalus in a kaolin-induced hydrocephalus mouse model in C57BL/6J mice [96]. Subsequent research by the same group suggested a link between pro-inflammatory microglial polarization and UPR^mt^ [97]. ATF5 functions as an upstream regulator that mediates the mitochondrial integrated stress response (ISR). The ATF5–UPR^mt^ axis plays a vital role in facilitating the repair of impaired mitochondria and upholding protein homeostasis within the cellular environment [98]. Specifically, they found that microglia exposed to lipopolysaccharide stimulation exhibited an upregulation of ATF5-dependent UPR^mt^, which significantly restrained the production of pro-inflammatory cytokines, including TNF-α, IL-6, and IL-1β [97]. This implies that ATF5-dependent UPR^mt^ may serve as a regulatory mechanism to dampen the inflammatory response in microglia and potentially mitigate neuroinflammation-associated damage. Xie et al. found that the activation of UPR^mt^ attenuated neuroinflammation after ischemic stroke [99], and in SAH UPR^mt^ regulated the neuronal mitochondrial homeostasis, which decreased the early brain injury [100].

Moreover, mitochondrial fission is a complex process that entails the division of a single mitochondrion into two separate mitochondria [101]. Prior research has established that the excessive activation of mitochondrial fission is a contributing factor to brain injury following a stroke [102]. However, there is evidence suggesting a neuroprotective effect of mitochondrial fission through the elimination of impaired mitochondrial segments, facilitation of signal transduction, enhancement of ATP production, and stabilization of mitochondrial DNA (mtDNA) [101]. In a study conducted by Busceti et al., wherein selective overexpression of uncoupling protein-2 (UCP2) was carried out in the corpus striatum of stroke-prone spontaneously hypertensive rats, they observed an upregulation of OPA1 and Fis1 [103]. OPA1 is associated with mitochondrial fusion, while Fis1 is related to mitochondrial fission, both processes occurring in the striatum tissue [103]. While the exact causality was not explicitly established, this experiment suggested that UCP2 may exert a neuroprotective effect by modulating mitochondrial fusion and fission.

Furthermore, appropriate mitophagy is instrumental in the improvement of stroke. Mitochondrial stress induces mitophagy to remove impaired mitochondria. In response to the pathological condition, the impaired mitochondrion is divided into two parts, one of which is functional segment, and anther of which undergoes dysfunction. Subsequently, the dysfunctional mitochondrial part is eliminated by mitophagy [104].

#### In ischemic stroke

The ATP depletion and Ca^2+^ overloading due to the deletion of oxygen and glucose induce the ISR that the proteins located in mitochondria-associated ER membrane (MAMs) are activated or translocate from ER to MAMs to maintain Ca^2+^ homeostasis and function as the assembly platform for inflammasomes in the brain when responding to the ischemic condition [105]. Appropriate activation in MAMs contributes to ischemic stroke improvement via regulating neuroinflammation.

FUNDC1 and Voltage Dependent Anion Channel 1 (VDAC1) are two of the MAMs protein. Mitochondrial VDAC1 has been determined to mediate inflammation activation in ischemic stroke. VDAC1 is the key mitochondrial protein that mediates the release of mtDNA in mitochondrial stress. In ischemia, the interaction of VDAC1 with Bax causes Cytochrome C release to the cytoplasm, which subsequently trigger mitochondrial permeabilization to evoke NLRP3 inflammasome assembly and cytokine expressions, including TNF-α, IL-6, IL-12 and interferon α/β [106]. Importantly, the lysine lactylation of VDAC1 is inhibited in neurons during ischemia [107], which contributes to the changes in metabolic phenotype and macrophage polarization. FUNDC1 can sense mitochondrial stress via OPA1 and then activate mitophagy [74]. Mitochondrial stress due to ischemic stroke results in FUNDC1 translocation from ER to mitochondria. In ischemic condition, FUNDC1 holds the stronger binding to LC3B that is required for mitophagy activation [108, 109]. Mitophagy is used to remove impaired mitochondria that promote neuroinflammation [110]. Thus, mitochondrial stress modulates neuroinflammation via the co-ordination with FUNDC1-mediated mitophagy.

In addition to the role of MAMs-related ISR, ATF4 also plays the protective role in stroke-associated neuroinflammation. As mentioned above, mitochondrial stress is capable of resisting neuroinflammation via inducing mitophagy. When UPR^mt^ is activated by ischemic condition, ATF4 can increase Parkin expression to cause mitophagy activation that has the ability to inhibit NLRP3 inflammasomes in the brain [111, 112]. Also, ATF4 is involved in the expressions of Bax, Bcl-2 and ER stress-related genes during ischemic stroke [113]. HSF1, another member of UPR^mt^, has been found to be related to ischemia disease pathogenesis [114]. After ischemia, HSF1 is highly enriched in the nuclear of P7 neurons and partly in microglia [115]. HSF1 has the capability to modulate the activation in microglia and astrocytes. HSF1 inhibits A1 astrocyte (a phenotype for neurotoxicity and neuroinflammation) through repressing MAPK/NF-κB pathway [116]. In microglia, HSF1 modulates gene expression that affect cell activation and inflammatory cytokines. For example, Liao et al. showed HSF1 could mediate the expression of miR-214-3p and nuclear factor of activated T cells 2 via its activity of transcription factor, thereby inhibiting microglia-mediated neuroinflammation [117]. Furthermore, dietary intake of n-3 polyunsaturated fatty acids attenuated mitochondrial oxidative stress and increased the mitophagy of astrocytes in the condition of hypoxia to limit A1-specific astrocyte polarization, thereby reducing the production of inflammatory cytokines and neurotoxins, subsequently improving the neurological outcomes of mice with ischemic stroke [118]. In addition, Sirtuin-3 (Sirt3), a NAD+-dependent deacetylase, was reported to modulate the UPR^mt^ and attenuate neuroinflammation after cerebral ischemia [99]. Sirt3 is a mitochondrial NAD+-dependent protein deacetylase that regulates the balance of energy metabolism, ROS generation [119]. Sirt3 activates the UPR^mt^ by increasing Foxo3a and Sphkl expression, subsequently Sphkl expression relieves neuroinflammation after ischemic stroke [99]. Thus, activation of UPR^mt^ and mitophagy reveal neuroprotection by mitigate neuroinflammation.

#### In hemorrhagic stroke

We have mentioned that mild mitochondrial stress activates mitophagy to eliminate neuroinflammation in ischemic stroke. In fact, this important mechanism is triggered as well as in hemorrhagic stroke. A report focusing on SAH in rats suggested mitophagy contributed to the inhibition to oxidative stress and neuron death [110]. Animal experiments showed mitophagy was activated at 24 h after hemorrhagic stroke modeling, with the increases of mitophagy biomarkers including Beclin-2, LC3B and PINK1 [120, 121]. Proteins in mitochondrial stress contributes to the mitophagy activation at this stage. In a study conducted on a SAH rat model, the administration of melatonin was found to reduce ROS levels by upregulating mitophagy [121, 122]. At the 24-h mark after SAH, melatonin treatment led to increased levels of mitophagy-associated proteins, specifically PINK and Parkin. Optineurin (OPTN) a protein can combine with ubiquitinated cargo contributing to mitophagy. OPTN is also associated with PINK1/Parkin-mediated mitophagy [123], which upregulates mitophagy and decreases NLRP3 activation in ICH [124]. This intervention resulted in a decrease in ROS content, mitigated morphological alterations in mitochondria, and ultimately inhibited the activation of the NLRP3 inflammasome [121]. In a rat SAH model, VDAC1 was found to be time-dependently upregulated within 48 h after SAH modeling, which induced mitophagy [125]. VDAC1 promoted mitophagy to reduce DAMPs and inhibit the death in neurons [125]. The role of VDAC1 in mitophagy may involve Ant1, an mPOS-related protein. The complex of VDAC1 and Ant1 drove mitophagy via causing MMP depolarization [126]. Importantly, Ant1 mediates the stability of PINK1 during mitophagy. Zoltan Arany et al. reported Ant1 promoted PINK1 accumulation on OMMs via closing TIM23-mediated PINK1 translocation in the pathological condition [127].

As described above, in mitochondria Sirt3 is a switch of mitochondrial energy regulation [128]. Activation of Sirt3, which promotes UPR^mt^, attenuates neuroinflammation and mitigates ATP, ROS and mitochondrial complex1 release after ICH [129, 130]. FGF21 functions as the mitokine that transmits stress signals for non-autonomous response. A clinical report determined serum FGF21 is the prognosis biomarker of ICH [131], suggesting its key role in hemorrhagic stroke. FGF21 has found to suppress the production of NF-κB-related cytokines during hemorrhagic disease. Thus, FGF21 has the potential to modulate neuroinflammation in hemorrhagic stroke. GDF15 is also the core mitokine for mitochondrial stress. GDF15 is significantly upregulated in patients with intracerebral and SAH [132]. However, the mechanism of GDF15 in hemorrhagic stroke has been not clearly described. Perhaps, GDF15 modulates cerebral microenvironment via GFRAL–RET heterodimer expressed in the hindbrain [133].

### Continuous and severe mitochondrial stress aggravates neuroinflammation during stroke

Stroke pathogenesis involves mitochondrial stress [12]. Neuronal toxicity leads to mitochondrial impairment, including MMP reduction, the increase of protein aggresomes and the dysfunction in UPR^mt^ [13]. Mitochondrial stress is the key event for quantifying control in mitochondria [12]. However, the continuous performance of mitochondrial stress may fail to restore mitochondrial homeostasis and induces the severe mitochondrial injury [134]. Mitochondrial stress responses become the cooperator of inflammation to induce the death in neurons during stroke when they are not enough to resist mitochondrial dysfunction in the pathological condition [13]. Mitochondrial stress affects glia activity. In this part, we review the reports showing the co-operation of severe mitochondrial stress and neuroinflammation in stroke, and describe the intriguing mechanism of severe mitochondrial stress in neuroinflammation based on common glia.

#### In ischemic stroke

Misfolded proteins are the main cause of secondary brain injury in ischemic stroke [13]. UPR^mt^ is used to decrease misfolded mitochondrial proteins and alleviate the pressure of protein import into mitochondria. However, this response is not enough to inhibit the sustained accumulation of misfolded proteins in ischemic condition. The UPR^mt^-related protein ATF4 has been determined to be the pro-death transcription factor promoting the progression of ischemic stroke although it plays neuroprotective role in the early stage of ischemic stroke. Overexpression of ATF4 in neurons contributes to the susceptibility to oxidative death. Thus, ATF4 has the harmful effect on neurons undergoing ischemic condition. The mouse model of ischemic stroke with ATF4 deletion shows the resistance to the oxidative stress-induced death in neurons [135]. In cerebral ischemia, the ectopic expression of ATF4 promoted JUNB and ETS1 via the JMJD3-dependent demethylation of H3K27m3, which aggravated neuroinflammation and neuronal death in the brain [136].

Ischemic stroke pathogenesis causes abundant mitochondrial stressors in the central nervous system (CNS). Then, mitochondrial stress leads to the massive release of mitochondrial contents such as mtDNA and mtROS, which promotes pro-inflammatory cytokine production and immunocyte activation during ischemic stroke [12]. Sirt6 is a NAD+-dependent protein deacetylase and ribosylation enzyme, which mediated inflammatory cytokine secretion and ROS production [137]. Overexpression of Sirt6 in endothelial lessen ischemic region by reduce mtROS and oxidized mtDNA, meanwhile decrease TNF-α, IL-18 and IL-1β [22]. A laboratory study based on lipopolysaccharide-stimulated macrophage suggested mtROS activated the deubiquitination of NOD-like receptor thermal protein domain associated protein 3 (NLRP3) to induce NLRP3 inflammasomes activation [138]. NLRP3 inflammasomes commonly expressed in the CNS when ischemic stroke occurs are positively correlated to the cerebral infarct and neurological deficit during stroke [139]. It is instrumental in inhibiting neuron death and neurological deficit during ischemic stroke to remove mtROS by exogenous treatment [140, 141].

Glia positively contribute to neuroinflammation in ischemic stroke. Microglia are the most important immune cell in CNS. Microglia activation following ischemia release inflammatory cytokine which aggravates neuroinflammation. Mitochondrial fission, the most important pathway of mitophagy [142], in microglia was associated with neuroinflammation after cerebral ischemia. Mounting evidence shows that the Janus kinase (JAK) and signal transducers and transcription (STAT) were activated in inflammation [143–145]. Dynamin-related protein 1 (Drp1) is a multidomain GTPase provoke mitochondrial fission, which is modulated by JAK2/STAT3 signaling [146]. Thus, activation of JAK2/STAT3 upregulates Drp1 expression then promotes mitochondrial fission in microglia and mediates neuroinflammation. Impaired mitochondria in neurons can evoke astrocyte activation via a crossing cellular pathway [147]. Astrocyte plays dual roles in ischemic stroke. Activated astrocytes support neurological restoration, whereas they also produce pro-inflammatory cytokines to aggravate neuroinflammation in ischemic stroke progression [148]. The sustained signals from impaired mitochondria result in a large amount of cytokine by activated astrocytes, leading to the continuous inflammatory microenvironment in the CNS. Similar to astrocytes, the inflammatory microglia also are maintained by severe mitochondrial stress, which is one of the major causes of inflammation infiltration into the brain during ischemic stroke. Oligodendrocytes are more susceptible to the stress than other types of glia. The activity and morphology of oligodendroglial mitochondria are unique. The differentiation from oligodendrocyte progenitor cells (OPCs) to oligodendrocytes creates the high demand of mitochondria during myelin biosynthesis while OPCs rely less on mitochondria-mediated ATP metabolism after myelination [149]. OPCs positively regulate the sustained inflammation in the brain. Netrin-1 expressed in oligodendrocytes monitors the subcellular location and recruitment of mitochondria [150]. Mitochondria tend to induce oligodendroglial apoptosis in the pathological condition such as ischemia, resulting in myelin loss and neurodegeneration.

#### In hemorrhagic stroke

Mitochondria are the main source of ROS after hemorrhagic stroke. The animal experiment related to SAH showed ROS burst due to the stress-induced opening of mitochondrial permeability transition pore [151]. As described above, mtROS functions as the communication molecule of mitochondrial stress. Cells start the downstream pathway when sensing mtROS. For example, excessive mitochondrial fission was observed in ICH [11]. Suppression of excessive mitochondrial fission seems to contribute to reduce NLRP3 and pro-inflammatory including IL-1β, IL-18, consequently, attenuate neuronal pyroptosis in ICH [152]. MtROS and mtDNA have been found to play the key role in the activation of NLRP3 inflammasomes. NLRP3 inflammasomes mediate the occurrence and development of pyroptosis that is an inflammatory death in cells and accompanied by the massive release of inflammatory cytokines. NLRP3 inflammasomes were increased in the microglia of mice with hemorrhagic stroke. Impaired mitochondria can activate NLRP3-mediated pyroptosis through mtROS release, which further enhances neuroinflammation and aggravates neuronal injury in hemorrhagic stroke [120, 121, 153].

ATF4, similar to its role in ischemic stroke, becomes a significant target of inhibiting neuronal death in several rodent models of hemorrhagic stroke [154]. Liu et al. identified ATF4 as the differentially expressed gene of ICH and predicted its function was involved in TNF-mediated inflammation [155]. They subsequently determined that ATF4 was upregulated in the rat model of cerebral hemorrhage, and found ATF4 downregulation was instrumental in inhibiting inflammation. Min et al. reported the activated microglia-derived exosomes improved necroptosis in neurons undergoing ICH via reducing ATF4 [156]. In mice with ICH, activated GCNF-eIF2α pathway inhibited ATF4 activity to reduce inflammation infiltration into the CNS [157].

In hemorrhagic stroke-induced neuronal injury, UPR^mt^ dysfunction is associated with the decrease in the complex of nucleotide exchange factor and HSP70. A study focusing UPR^mt^ in hemorrhagic stroke showed a mechanism between GrpE like 1, mitochondrial (GrpEL1), a nucleotide exchange factor, and mitochondrial HSP70 (mtHSP70). Although mtHSP70 upregulation was observed in oxyhemoglobin-induced cell model of hemorrhagic stroke, the constitutive binding of GrpEL1 and mtHSP70 was decreased, which resulted in the UPR^mt^ inhibition that contributed to mitochondrial dysfunction and impairment [100]. Obviously, the sustained pathological condition in neurons contributes to the overaccumulation of mitochondrial stress response. The sustained mitochondrial stress further impairs mitochondrial genome [158], which may serve as the major cause of mitochondrial dysfunction in hemorrhagic stroke.

## Conclusion

In summary, disruptions in mitochondrial homeostasis serve as an initial event contributing to NVU dysfunctions and play a pivotal role in the pathophysiology of stroke. The synergy between neuroinflammation and proper mitochondrial stress can effectively eliminate harmful stimuli and facilitate neurological recovery. Conversely, severe mitochondrial stress exacerbates neuroinflammation in the context of stroke. Such proper mitophagy appears to offer neuroprotection by eliminating damaged mitochondria, while excessive mitophagy can detrimentally impact energy production and mitochondria-associated signaling pathways. Consequently, inhibiting mitochondrial stress emerges as a potential and unique therapeutic approach for stroke. Key proteins involved in mitochondrial stress, such as ATF5, ATF4, HSF1, FGF21, and GDF15, hold promise as potential targets for modulating neuroinflammation during stroke.

Despite significant advancements in understanding the mechanisms underlying mitochondrial dynamics and mitophagy following stroke, this field remains complex and necessitates further investigation. The precise effects of mitophagy in the context of stroke demand further exploration, as they hold the potential to yield valuable insights into stroke treatment strategies. Techniques such as genome, transcriptome, proteome, and epigenome sequencing can uncover molecular heterogeneity, offering patient-specific insights into MQC and potentially paving the way for novel therapeutic interventions in stroke.

## Data Availability

Not applicable.

## Abbreviations

BBB: Blood–brain barrier
DAMPs: Damage associated molecular patterns
OMM: Outer mitochondrial membrane
IMM: Inner mitochondrial membrane
OXPHOS: Oxidative phosphorylation
HSP: Heat shock protein
TOM: Translocase of the outer membrane
MTS: Mitochondrial targeting pre-sequences
MMP: Mitochondrial membrane protein
GSDMD: Gasdermin D
PGC-1α: Peroxisome proliferator-activated receptor γ coactivator 1α
NF-κB: Nuclear factor kappa B
SOD2: Superoxide dismutase 2
GPX1: Glutathione peroxidase 1
UPR^mt^: Mitochondrial unfolded protein response
ETC: Electron transport chain
CHCHD10: Coiled-coil-helix-coiled-coil-helix domain-containing 10
ISR: Integrated stress response
mPOS: Mitochondrial precursor overaccumulation stress
mitoCPR: Mitochondrial compromised protein import response
ATFS1: Activating transcription factor associated with stress 1
NLS: Nuclear localization signal
HSF1: Heat shock transcription factor 1
mtPO: Mitochondrial precursor overaccumulation
HRI: Heme-regulated inhibitor
eIF2α: Eukaryotic initiation factor 2α
NVU: Neurovascular unit
CNS: Central nervous system
OPCs: Oligodendrocyte progenitor cells
UCP2: Uncoupling protein-2
MAMs: Mitochondria-associated ER membrane
VDAC1: Voltage-dependent anion channel 1
